# Point of sampling detection of Zika virus within a multiplexed kit capable of detecting dengue and chikungunya

**DOI:** 10.1186/s12879-017-2382-0

**Published:** 2017-04-20

**Authors:** Ozlem Yaren, Barry W. Alto, Priyanka V. Gangodkar, Shatakshi R. Ranade, Kunal N. Patil, Kevin M. Bradley, Zunyi Yang, Nikhil Phadke, Steven A. Benner

**Affiliations:** 1 Foundation for Applied Molecular Evolution (FfAME), Gainesville, FL USA; 2 Florida Medical Entomology Laboratory, University of Florida, Vero Beach, FL USA; 3 GenePath Dx (Causeway Healthcare), Pune, Maharashtra India; 4 Firebird Biomolecular Sciences LLC, Alachua, FL USA

**Keywords:** Point-of-care diagnostics, Multiplexed isothermal amplification, Zika detection, Fluorescence read-out, Sample preparation, Mosquito surveillance, Virus detection

## Abstract

**Background:**

Zika, dengue, and chikungunya are three mosquito-borne viruses having overlapping transmission vectors. They cause diseases having similar symptoms in human patients, but requiring different immediate management steps. Therefore, rapid (< one hour) discrimination of these three viruses in patient samples and trapped mosquitoes is needed. The need for speed precludes any assay that requires complex up-front sample preparation, such as extraction of nucleic acids from the sample. Also precluded in robust point-of-sampling assays is downstream release of the amplicon mixture, as this risks contamination of future samples that will give false positives.

**Methods:**

Procedures are reported that directly test urine and plasma (for patient diagnostics) or crushed mosquito carcasses (for environmental surveillance). Carcasses are captured on paper samples carrying quaternary ammonium groups (Q-paper), which may be directly introduced into the assay. To avoid the time and instrumentation requirements of PCR, the procedure uses loop-mediated isothermal amplification (LAMP). Downstream detection is done in sealed tubes, with dTTP-dUTP mixtures in the LAMP with a thermolabile uracil DNA glycosylase (UDG); this offers a second mechanism to prevent forward contamination. Reverse transcription LAMP (RT-LAMP) reagents are distributed dry without requiring a continuous chain of refrigeration.

**Results:**

The tests detect viral RNA in unprocessed urine and other biological samples, distinguishing Zika, chikungunya, and dengue in urine and in mosquitoes infected with live Zika and chikungunya viruses. The limits of detection (LODs) are ~0.71 pfu equivalent viral RNAs for Zika, ~1.22 pfu equivalent viral RNAs for dengue, and ~38 copies of chikungunya viral RNA. A handheld, battery-powered device with an orange filter was constructed to visualize the output. Preliminary data showed that this architecture, working with pre-prepared tubes holding lyophilized reagent/enzyme mixtures and shipped without a chain of refrigeration, also worked with human plasma samples to detect chikungunya and dengue in Pune, India.

**Conclusions:**

A kit, complete with a visualization device, is now available for point-of-sampling detection of Zika, chikungunya, and dengue. The assay output is read in ca. 30 min by visualizing (human eye) three-color coded fluorescence signals. Assay in dried format allows it to be run in low-resource environments.

**Electronic supplementary material:**

The online version of this article (doi:10.1186/s12879-017-2382-0) contains supplementary material, which is available to authorized users.

## Background

Zika virus (genus *Flavivirus*, family *Flaviviridae*) is native to Africa and consists of one Asian and two African genetic lineages [1, 2]. Up until the last decade, Zika virus predominantly circulated in a zoonotic cycle involving forest-dwelling *Aedes* mosquitoes and non-human primates in Africa and Asia.

Identified in 1947, Zika infections in humans remained sporadic for ~50 years before emerging in the Pacific and the Americas [3]. An outbreak of Zika fever occurred on Yap in the Federated States of Micronesia in 2007, and then in French Polynesia in 2013 and 2014. In 2015, Zika virus emerged for the first time in Brazil. It has now spread rapidly throughout the Americas along with the chikungunya virus, an *alphavirus* and dengue virus, another *flavivirus*. The emergence of Zika outside of Africa has been associated with a change in transmission from a predominantly zoonotic cycle to a transmission cycle involving human hosts and domestic mosquito vectors, including *Aedes aegypti* and *Aedes albopictus*. These invasive *Aedes* species share similar ecology and are primary vectors of chikungunya and dengue viruses as well [4, 5].

The clinical presentation of Zika fever is nonspecific and can be misdiagnosed, as symptoms of Zika are similar to other mosquito-spread viruses like chikungunya and dengue. A majority of cases are asymptomatic (80%, according to the CDC [6]). In other cases, illness is clinically mild with symptoms lasting from several days to a week, including fever, rash, joint pain, conjunctivitis, myalgia, and headache. Serious illnesses associated with Zika virus include Guillain-Barré syndrome in adults, microcephaly in neonates, and chronic musculoskeletal diseases that may last months to years [7]. Two cases are reported from New Caledonia having co-infection of Zika and dengue; Colombia reports one patient co-infected with Zika, chikungunya and dengue. This makes differential diagnosis even more challenging [8].

Since specific treatment or an approved vaccine is currently unavailable, rapid and reliable detection of Zika is needed for initiation of control and preventive measures, such as mosquito control and patient management. Standard serological approaches, such as antibody detection and immunoassays, often have inadequate sensitivity. Further, they are complicated by cross-reactivity in patients who have previously been infected by other *flaviviruses* from the endemic region [4]. Therefore, nucleic acid-targeted diagnostics remain as the best means to detect and differentiate Zika, chikungunya and dengue.

Biological confirmation of Zika, chikungunya and dengue infections is generally based on detection of viral RNA in blood by using reverse transcription PCR (RT-PCR) or real-time RT-PCR combined with hydrolysis probes (e.g. TaqMan probes). In several studies, however, patients were found to give positive tests for Zika in their saliva and urine, but not blood [9, 10]. Thus, urine and saliva samples for Zika detection are preferred over blood because of higher viral titers and prolonged presence of virus [11, 12]. Even though blood samples are shown to have higher viral loads for chikungunya and dengue, urine and saliva samples can still be used to diagnose these diseases, especially desirable for their easy collection and handling [13, 14].

RT-PCR diagnostics is considered the gold standard for diagnostics. However, it requires extensive sample preparation and expensive equipment to control heating and cooling cycles. This means that PCR tests must generally be performed at specialized facilities. A point-of-sampling nucleic acid test would be valuable if it relied on isothermal amplification rather than PCR. This test could be used in lower resource areas, including college infirmaries, doctor’s offices, airport clinics, ambulances, and forward-deployed military units.

**Fig. 1:**
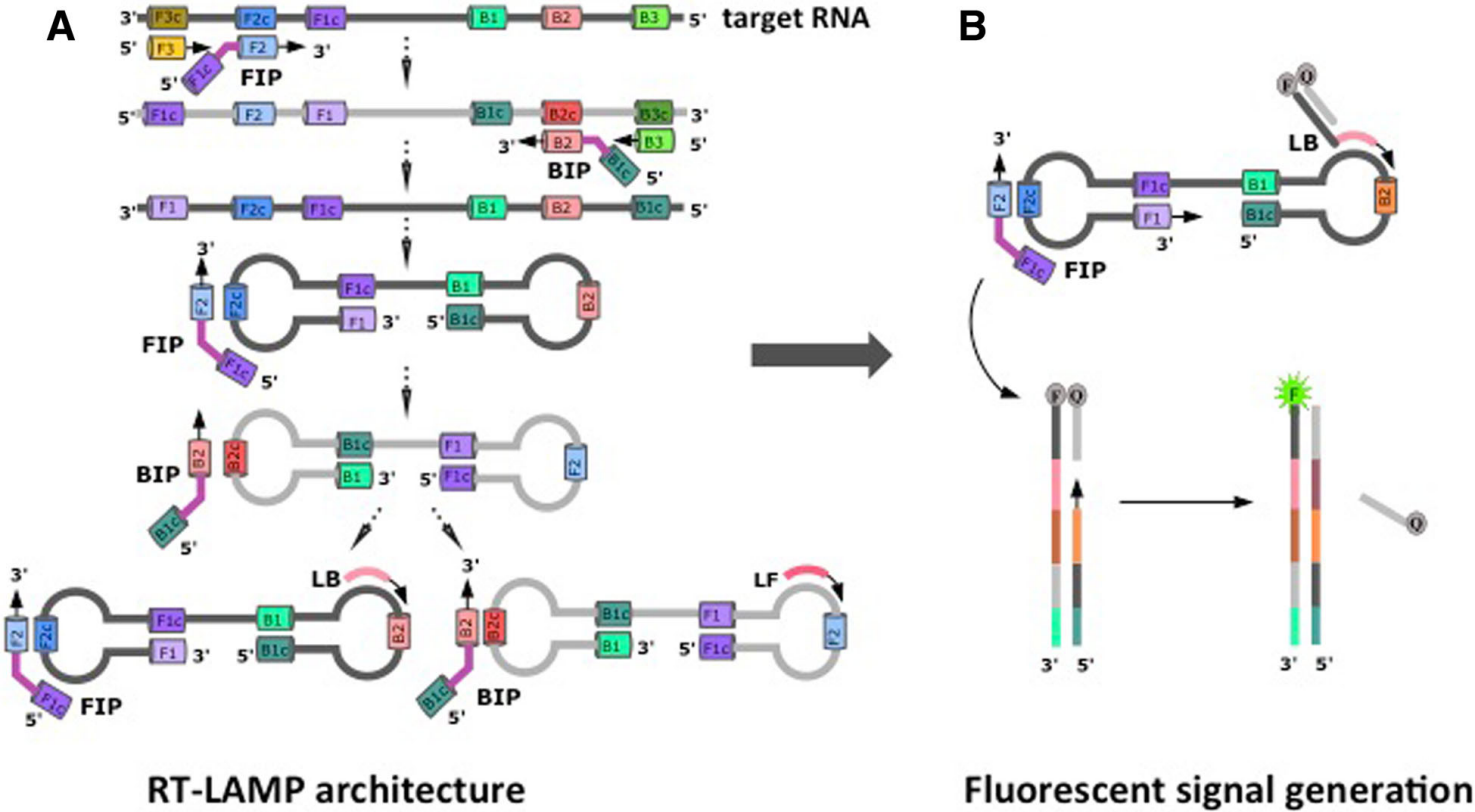
**a** RT-LAMP is initiated by adding internal primers (FIP or BIP) that annealed by Watson-Crick complementarity to regions (F2c or B2c) within the target RNA. The outer primer (F3 or B3) then hybridizes to its priming site (F3c or B3c) on the target RNA and initiates the formation of self-hybridizing loop structures by strand invasion of the DNA sequences already extended from the internal primers (FIP and BIP), resulting in a dumbbell structure. RT-LAMP process can be accelerated by loop primers (LF and LB). **b** Further, priming region of the fluorescently tagged probe (e.g. LB) is extended by a strand-displacing polymerase, and primer extension from the reverse primers then reads through the primer on the fluorescently tagged probe, displacing the probe that bears a quencher moiety. This separates the fluorescently tagged oligonucleotide from the quencher tagged probe, allowing the fluorescence to be observed in real-time and measured from fluorescently tagged probe that has been incorporated into RT-LAMP products

A powerful RT-PCR alternative, reverse transcription loop-mediated isothermal amplification (RT-LAMP) usually employs a set of six primers that bind to eight distinct regions within the target RNA. It runs at constant temperature, usually between 60 °C and 70° **(**Fig. 1a **)**. [15]. During the initial stages of RT-LAMP, the F2 region of FIP hybridizes to F2c region of the target RNA, and reverse transcriptase initiates the synthesis of the complementary DNA strand. Outer Primer F3 hybridizes to the F3c region of the target RNA and extends, displacing the FIP linked strand. This displaced strand forms a loop at its 5′-end. Then, the single stranded DNA with a loop at the 5′ end serves as a template for the internal BIP primer, whose B2 portion hybridizes to B2c region of the template DNA.

DNA synthesis is then initiated by a strand-displacing polymerase leading to the formation of a complementary strand and opening of the 5′-end loop. The outer primer B3 then hybridizes to B3c region of the target DNA and is extended, displacing the BIP-linked strand. This results in the formation of a dumbbell shaped DNA. The dumbbell structure then becomes a seed for exponential LAMP amplification. This amplification is further accelerated by the loop primers (LF and LB), which are designed to hybridize between F1c and F2, B1c and B2, respectively [16]. The amplification products include concatemers of the region in the analyte that is targeted, and may fold to a form “cauliflower-like structures”, which have multiple and repeating loops.

Although Zika detection using RT-LAMP architecture has been previously reported, these methodologies are based on a single target detection, signal generation is not sequence-specific (e.g. turbidity measurement or use of dsDNA binding dyes), lacks multiplexing ability and can be deceived by off-target amplifications, therefore susceptible to creating false positives [17–19].

To enable multiplexing and real-time monitoring, we have coupled target-specific fluorescently tagged strand displaceable probes with RT-LAMP to detect Zika, chikungunya and dengue viruses in biological samples such as urine and plasma, and mosquito carcasses infected with Zika and chikungunya viruses.

## Methods

### Laboratory setting

Virus propagation and mosquito infection studies were performed at BSL-3 facility of the Florida Medical Entomology Laboratory in Vero Beach, FL. RT-LAMP experiments were performed in the BSL-2 laboratory shared by FfAME and Firebird Biomolecular Sciences LLC in Alachua, FL. Patient samples (whole blood collected in EDTA) suspected with chikungunya or dengue were centrifuged to separate plasma in GenePath Dx facility (Causeway Healthcare, Pune, India). All samples that were used for RT-LAMP have previously been tested positive by quantitative PCR.

### Primers and probes

Primer design was performed using in-house software, OligArch v2 (FfAME, Alachua, FL), designed to create primer sets that account for the evolutionary variation within the genomes of viral targets. Viral sequences for dengue-1 were downloaded from the Broad Institute [20], while those for other targets were downloaded from the NIAID Virus Pathogen Database and Analysis Resource (ViPR) [21]. Multiple sequence alignments (MSAs) were created for these sequences using MUSCLE v3.8.31 [22]. The resulting MSAs were used as input to OligArch, which searches for primer sets that are conserved within a target of interest while avoiding unintended targets also included within the MSA (allowing, for example, distinction between dengue subtypes).

Rules for LAMP design were followed using criteria from the Eiken Genome website [23]. Designed LAMP sets were compared to the NCBI RNA virus database using NCBI BLAST [24] to eliminate sets that would cross-react. Sets were further compared, using in-house software PrimerCompare v1 (FfAME, Alachua, FL), to eliminate sets with primers that would dimerize in a multiplexed assay to produce the final sets of LAMP primers.

LAMP primers and strand displaceable probes were purchased from Integrated DNA Technologies (IDT, Coralville, IA) (Table 1). Strand-displaceable probes were 5′-labeled with FAM, HEX, TAMRA and TET for Zika, chikungunya, dengue-1 and mitochondrial DNA (positive control), respectively. Quencher probes which partially complementary to the fluorescently labeled probes was 3′-labeled with IowaBlack-FQ. Alternatively, probes targeting *Ae. aegypti* small subunit ribosomal RNA (SSU rRNA) were 5′-labeled with IowaBlack-FQ and 3′-labeled with FAM. Double strand portion of the probes were screened against any viral genome sequence and mosquito genomic sequence (see Additional file 1: Table S1 for additional primers and probes as positive controls**)**.

**Table 1:** Primers and strand displaceable probes

Target virus	Name	Sequence (5′-3′)	Length	Start Pos	End Pos
Zika	ZV-F3	GAGACTGCTTGCCTAG	16	9905	9920
	ZV-B3	CTGGGGTCTTGTCTTC	16	10,145	10,130
	ZV-LF	CAGTTGGAACCCAGTCAAC	19	10,028	10,010
	ZV-LB	GTGGAACAGAGTGTGGATTG	20	10,093	10,112
	ZV-FIP	CCATGGATTGACCAGGTAGTTTTTTCGACTGATGGCCAATG	41	9974	10,053
	ZV-BIP	ACCACTGARGACATGCTTGTTTTTCATGTGGTCGTTYTCC	40	10,070	10,129
	ZV-LB_NatTail	**FAM**-CGGGTTTGCGCTCAGCCATCCGTTCAGTCCGTCAGGTCAG-GTGGAACAGAGTGTGGATTG	60	10,093	10,112
Chikungunya	CH-F3	CGTCAACGTACTCCTAAC	18	2891	2908
	CH-B3	ACGTTGGCTTTRTTTTGG	18	3094	3077
	CH-LF	AGCGTCTTTATCCACGGG	18	2968	2951
	CH-LB	AYGCATCRATAATGGCGGG	19	3025	3043
	CH-FIP	GAAGTTTCCTTTCGGTGGGTTTTTGGAAGACACTYTCYGG	40	2932	2993
	CH-BIP	AAGGAGTGGGAGGTGGATTTTTTCAYTTGGTGACTGCAG	39	3006	3063
	CH-LF_NatTail	**HEX**-CGGGTTTGCGCTCAGCCATCCGTTCAGTCCGTCAGGTCAG-AGCGTCTTTATCCACGGG	58	2968	2951
Dengue-1	D1-F3	ACAGCYCTGAATGAYATGG	19	9583	9601
	D1-B3	GCAGTTTCTCTCAGGC	16	9803	9788
	D1-LF	CACTTGYTGCCARTCATTCC	20	9666	9647
	D1-LB	CCATGCCGYAACCAAG	16	9727	9742
	D1-FIP	CTGGTGGAARTGGTGTGATTTTTTGGGAACCTTCAAAAGG	40	9628	9693
	D1-BIP	GAAGGAYGGGAGGGAAATAGTTTTTTTAGCCCTRCCCACAAG	42	9702	9763
	D1-LB_NatTail	**TAMRA**-CGGGTTTGCGCTCAGCCATCCGTTCAGTCCGTCAGGTCAG-CCATGCCGYAACCAAG	56	9727	9742
Mitochondrial DNA	MtDNA-F3	AGCCTACGTTTTCACAC	17	9183	9199
	MtDNA-B3	GCGCCATCATTGGTAT	16	9410	9395
	MtDNA-LB	GCCTAGCCATGTGATTTCAC	20	9322	9341
	MtDNA-LF	GGCATGTGATTGGTGGGT	18	9254	9237
	MtDNA-FIP	GTCATGGGCTGGGTTTTACTTTTTCTACCTGCACGACAAC	40	9213	9228
	MtDNA-BIP	CTCAGCCCTCCTAATGACCTTTTTGAGCGTTATGGAGTGG	40	9359	9344
	MtDNA-LB_NatTail	**TET**-CGGGTTTGCGCTCAGCCATCCGTTCAGTCCGTCAGGTCAGGCCTAGCCATGTGATTTCAC	60	9322	9341
	Common quencher	CTGACCTGACGGACTGAACGGATGGCTGAGCGCAAACCCG-**Iowa Black FQ**	40		
*Aedes aegypti* SSU rRNA	Aae-F3	GGTGTAGTGTGACCTG	16	2501	2524
	Aae-B3	GCTAGCTAATGACCAGC	17	2883	2866
	Aae-LB	AAGGGCCGGGAAATCG	16	2777	2793
	Aae-LF	TCTAAGGGCATCACGGAC	18	2705	2687
	Aae-FIP	CGTGCAGCCCAGAACATTTTTGCAAAATGAGATTGAGCG	39	2660	2678
	Aae-BIP	CAACGCGTATCCTTGCCTTTTTAATCCCGACTAAATGCG	38	2820	2803
	Aae-LF_NatTail-5IB-FQ	**IowaBlack FQ-** GGGTTTGCGCTCAGCCATCCGTTCAGTCCGTCAGGTCAG TCTAAGGGCATCACGGAC	57	2705	2687
	Aae-LF_NatTail_comp FAM	CTGACCTGACGGACTGAACGGATGGCTGAGCGCAAACCC-**FAM**	39		

Underlined sequences are double strand segments of strand-displacing probes. FAM was used for Zika detection and positive control for *Ae. aegypti* ssu rRNA detection (λ_ex_-λ_em_ = 495 nm-520 nm, color observed with excitation at 470 nm, green), HEX was used for chikungunya detection (λ_ex_-λ_em_ = 538 nm-555 nm, color observed with excitation at 470 nm, yellow), TAMRA was used for dengue-1 detection (λ_ex_-λ_em_ = 559 nm-583 nm, color observed with excitation at 470 nm, orange), TET was used for mitochondrial DNA detection as positive control in urine (λ_ex_-λ_em_ = 522 nm-539 nm, color observed with excitation at 470 nm, yellow). IowaBlack-FQ was used as a common quencher with absorption range of 420-620 nm. Pos: position. SSU rRNA: small subunit ribosomal RNA

### Virus propagation and mosquito infection

Virus isolates included the following: Zika virus (Puerto Rico), two chikungunya viruses (La Réunion and British Virgin Islands) and dengue-1 (Key West, FL). All viruses were passaged 1-3 times in African green monkey kidney (Vero) cells and viral titers for Zika and dengue-1 were determined by plaque assay. Chikungunya viral RNA was quantitated using the Superscript III One-Step qRT-PCR with Platinum® Taq kit by Invitrogen (Invitrogen, Carlsbad, CA) as described previously [25] with the CFX96 Real-Time PCR Detection System (Bio-Rad Laboratories, Hercules, CA) (Table 2).

**Table 2:** Viruses studied

Virus, Strain (GenBank accession number)	Family/Genus	Viral titers
Zika virus (ZV), Puerto Rico (PRVABC59, KU501215.1)	Flaviviridae/Flavivirus Group IV, positive, ssRNA	2.85 × 10^8^ pfu/mL
Chikungunya virus (CH), British Virgin Islands (Asian lineage, KJ451624)	Togaviridae/Alphavirus Group IV, positive, ssRNA	2.42 × 10^8^ genomes/mL
Chikungunya virus (CH), La Reunion (Indian Ocean lineage, LR2006-OPY1, KT449801)	Togaviridae/Alphavirus Group IV, positive, ssRNA	1.89 × 10^8^ genomes/mL
Chikungunya virus (CH), La Reunion extracted total NA from *Aedes aegypti* female (Indian Ocean lineage, LR2006-OPY1, KT449801)	Togaviridae/Alphavirus Group IV, positive, ssRNA	3.85 × 10^5^ genomes/mL
Dengue serotype 1 (D-1), Key West (FL) (JQ675358)	Flaviviridae/Flavivirus Group IV, positive, ssRNA	1.22 × 10^6^ pfu/mL

pfu: plaque forming unit

Infection of *Ae. aegypti* females with Zika and chikungunya viruses was explained in detail in Additional file 1. Following infection, mosquito legs were separated from the body to confirm the infection. The legs were placed in a centrifuge tube with 1 mL media, two zinc beads, and homogenized at 25 Hz for 3 min (TissueLyser: Qiagen, Inc., Valencia, CA). The homogenate was then clarified by centrifugation for 10 min at 4 °C. RNA was then extracted from an aliquot of the mosquito leg homogenate (160 μL) using the QIAamp viral RNA mini kit (Qiagen, Valencia, CA) and eluted in TE buffer (50 μL) according to the manufacturer’s protocol. Viral RNA was detected using the Superscript III One-Step qRT-PCR with Platinum® Taq kit by Invitrogen (Invitrogen, Carlsbad, CA) with primers and probes specific to each virus (Integrated DNA Technologies) (Additional file 1: Table S2). Programs used for qRT-PCR were described elsewhere for Zika [2], chikungunya [26]. Leg viral titers were then determined by plaque assay (Table 3).

**Table 3:** Zika and chikungunya viral titers in the infected *Aedes aegypti* mosquito legs

Zika (ZV) Mosquito identity, Strain	Leg titer pfu/mL	Chikungunya (CH) Mosquito identity, Strain	Leg titer pfu/mL
ZV 3, Puerto Rico	2.54 × 10^3^	CH 320, La Reunion	1.78 × 10^4^
ZV 4, Puerto Rico	9.80 × 10^2^	CH 328, La Reunion	1.51 × 10^4^
ZV 7, Puerto Rico	5.10 × 10^3^	CH 191, La Reunion	2.41 × 10^3^
ZV 9, Puerto Rico	4.76 × 10^3^	CH 378, British Virgin Islands	3.21 × 10^5^
		CH 401, British Virgin Islands	4.52 × 10^4^

Pfu: plaque forming unit

### RT-LAMP procedure

Reaction mixtures (50 μL total volume) contained a 10X primer set (5 μL, 16 μM FIP and BIP, 2 μM F3 and B3, 5 μM LF (or LB for chikungunya), 2 μM LB (or LF for chikungunya), 4 μM LF quencher probe, and 3 μM LB-fluorescent probe (or LF probe for chikungunya)), deoxynucleoside triphosphates (dNTPs, 1.4 mM of each), Tris-HCl buffer (20 mM, pH 8.8), KCl (50 mM), (NH_4_)_2_SO_4_ (10 mM,) MgSO_4_ (8 mM), Tween® 20 (0.1%), DTT (1 mM), Bst 2.0 WarmStart® DNA Polymerase (16 U, NEB, Ipswich, MA), WarmStart® RTx Reverse Transcriptase (15 U, NEB, Ipswich, MA), and RNaseOUT™ recombinant ribonuclease inhibitor (80 U, Thermo Fisher Scientific, Waltham, MA). To this mixture was added extracted viral RNAs (1 μL, Zika, chikungunya or dengue-1). Samples were incubated at 65 °C for 45 min, then analyzed by agarose gel electrophoresis (2.5%) in 1X TBE buffer, followed by ethidium bromide staining, using an appropriate DNA size marker (50 bp ladder; Promega, Madison, WI).

For multiplexed RT-LAMP, each 10X primer set (5 μL each, Zika, chikungunya and dengue-1) was added in the same manner to RT-LAMP mixture (total 50 μL volume).

### RT-LAMP with urine samples

Initially, varying concentrations of urine (50% to 0%) were tested in RT-LAMP reactions. Typically, viral RNA spiked urine was included in the reaction mixture to a 10% final concentration without any purification step. As a positive control, mitochondrial DNA targeting LAMP primers were designed for use in urine [27]. Similarly, 10% saliva and plasma samples were also tested for Zika detection.

### Real-time RT-LAMP

For the real-time monitoring of RT-LAMP, the reactions were incubated at 65 °C for 60-90 min and the fluorescence signals from FAM-labeled probe for Zika (λ_ex_/λ_em_ = 495 nm/520 nm (using filter 483-533 nm), HEX-labeled probe for chikungunya (λ_ex_/λ_em_ = 538 nm/555 nm (using filter 523-568 nm), or TAMRA-labeled probe for dengue-1 (λ_ex_/λ_em_ = 559 nm/583 nm (using filter 558-610 nm) were recorded every 30 s using Roche Light Cycler 480 (Roche Life Sciences, Indianapolis, IN). Initial real-time LAMP experiments contained only 80 nM of fluorescently labeled LB or LF probe instead of 300 nM. Final primer concentrations in this set-up were as follows: 1.6 μM FIP and BIP, 0.2 μM F3 and B3, 0.4 μM LF and LB, 0.08 μM LB-fluorescent probe (or LF for chikungunya) and 0.2 μM quencher probe. Final primer concentrations with 300 nM strand displacing probes were as follows; 1.6 μM FIP and BIP, 0.2 μM F3 and B3, 0.5 μM LF (or LB for chikungunya), 0.2 μM LB (or LF for chikungunya), 0.3 μM LB-fluorescent probe (or LF for chikungunya) and 0.4 μM quencher probe.

Additionally, images of fluorescence generated by strand displaceable probes, induced by blue LED light (470 nm) at room temperature, were recorded through an orange filter by a cell phone camera (e.g. iPhone 6 s).

### Q-paper based RT-LAMP on mosquito samples

Quaternary ammonium modified paper (Q-paper) was made by treating Whatman filter paper (Grade 1) with an NaOH solution, followed by washing with water and then treatment with glycidyltrimethyl ammonium chloride, following a literature procedure [28]. The Q-paper sheets were cut into small squares (~0.5 cm^2^). *Aedes aegypti* female mosquitoes were crushed on each paper square with a micro pestle. The crushed carcasses were treated with aqueous NH_3_ (1 M, 100 μL, pH ≈ 12). The papers were washed once with 50% EtOH (100 μL) and once with ddH_2_O (100 μL), and air-dried. The paper squares, with and without target virus, were then placed inside RT-LAMP mixture and incubated 65 °C for 45 min. Prior to testing viruses, a primer set (as positive control) targeting *Ae. aegypti* SSU rRNA was tested on Q-paper crushed non-infected mosquito samples.

### Managing forward contamination

Carryover contamination was prevented by incorporation of dUTP by Bst 2.0 WarmStart® DNA Polymerase (NEB, Ipswich, MA) during RT-LAMP, and Antarctic thermolabile UDG (NEB, Ipswich, MA) was used to destroy DNA containing dU. Reactions were run with a 50% inclusion of dUTP mixed with dTTP giving final 0.7 mM dTTP, 0.7 mM dUTP, and 1.4 mM each dATP, dCTP and dGTP. Antarctic thermolabile UDG (1 μL, 2 units) was added to RT-LAMP reaction mixture (50 μL). Samples were first incubated at 25 °C for 5 min and then heated to 65 °C for 20-45 min.

### Lyophilization of RT-LAMP reagents

A mixture of Bst 2.0 WarmStart® DNA Polymerase (16 U), WarmStart® RTx Reverse Transcriptase (15 U), RNaseOUT™ (80 U) and Antarctic thermolabile UDG (2 U) in dialysis buffer (200 μL, 10 mM Tris-HCl pH 7.5, 50 mM KCl, 1 mM DTT, 0.1 mM EDTA10 and 0.1% Triton X-100) was placed in an ultrafiltration membrane (10 kDA cut-off limit, Millipore, Billerica, MA). Samples were centrifuged at 14,000 x g for ca. 15 min to concentrate (down to ~5 μL) and to remove glycerol. 10X LAMP primers (5 μL), dNTPs (10 mM each, 7 μL) and glycerol free enzyme mix (5 μL) were combined and lyophilized and supplemented with 1.1X-LAMP rehydration buffer (22 mM Tris-HCl, pH 8.8, 55 mM KCl, 11 mM (NH_4_)_2_SO_4_, 8.8 mM MgSO_4_, 0.11% Tween® 20, 1.1 mM DTT). Plasma samples (5 μL) were mixed with 1.1X rehydration buffer (45 μL) and incubated at 65 °C for 45 min. The resulting fluorescence signal was observed by blue LED excitation (470 nm) through an orange filter.

## Results

### Modifications on the architecture of RT-LAMP

Standard RT-LAMP architecture from Fig. 1a was modified to improve the signal detection and to support multiplexing. Here, an additional component was added in the form of a “strand displaceable probe”. This comprises two DNA strands that are complementary over part of their lengths. The first oligonucleotide strand has a quencher moiety at its 3′-end; the second DNA strand has a fluorophore covalently attached at its 5′-end. When the two strands are hybridized, the quencher and the fluorophore are brought into close proximity, and no fluorescence is observed. However, the 3′-portion of the second DNA strand is not covered by a hybridizing segment of the first DNA strand; left in a single stranded form, this is a priming sequence complementary to a segment of the loop region of the dumbbell structure created by the initial step of RT-LAMP, not on the target RNA itself.

Further, since the priming sequence hybridizes on the loop region, the signal is created only after the initial dumbbell is formed. Therefore, it cannot be created by any number of artifacts that are common in RT-LAMP. This duplex region is entirely under the control of the designer, and need not have any relation to any target sequence. Further, when multiplexing is applied, same sequence may be used with different fluorophore:quencher pairs.

During RT-LAMP, the priming region of the fluorescently tagged probe is extended by a strand-displacing polymerase **(**Fig. 1b **)**. Then, extension from the reverse primers reads through the primer on the fluorescently tagged probe, displacing the probe that bears the quencher moiety. This separates the fluorescently tagged oligonucleotide from the quencher tagged probe, allowing the fluorescence to be observed in real-time and measured from fluorescently tagged probe that has been incorporated into RT-LAMP products.

### Testing RT-LAMP primers

**Fig. 2:**
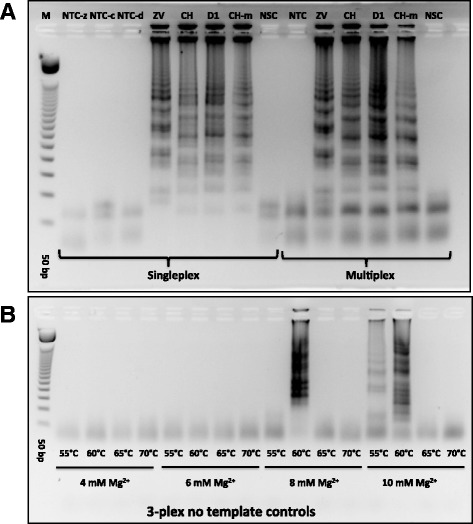
Gel electrophoresis analysis of RT-LAMP products. **a** Testing RT-LAMP primers for Zika virus (ZV), chikungunya (CH), and dengue serotype 1 (D-1) in 1-plex and 3-plex formats. M is a 50 bp DNA ladder, NTC-z/c/d are no template controls for ZV, CH and D-1 primers. CH-m was total nucleic acid pre-extracted from CH-infected *Ae. aegypti* female mosquito. NSC was designated as non-specific control where total nucleic acid extracted from non-infected *Ae. aegypti.* Viral titers used were as follows: 2.85 pfu for ZV, 242 genomic copies for CH, and 1.22 pfu for D-1. **b** Testing different RT-LAMP temperatures (55 °C to 70 °C) and Mg^2+^ concentrations (4 mM to 10 mM) in the presence of all three LAMP primers for ZV, CH and D-1 with no target RNA

Prior to sealed tube analysis, the performance of the RT-LAMP primers (Table 1) with the various virus targets as well as positive controls for urine and mosquito samples was assessed by gel electrophoresis. The samples were total RNA extracted from viral stocks cultured in African green monkey kidney (Vero) cells. In one case (for chikungunya), the viral RNA used was extracted using a “total nucleic acids” preparation kit from virus-infected mosquitoes (Table 2). This sample included, of course, mosquito RNA and DNA. Figure 2a shows some representative results with RT-LAMP performed with these samples. In both singleplexed and multiplexed cases, the yields of LAMP products, appearing in a gel as a ladder of concatemers, were similar. Negative control samples gave only the bands for primers themselves, including the non-specific target control where total nucleic acid extracted from healthy *Ae. aegypti* female mosquito was used as the template. To optimize LAMP conditions, magnesium concentrations and operation temperatures were varied. Higher magnesium concentrations and lower LAMP temperatures generated nonspecific amplicons. Therefore, 8 mM magnesium and 65 °C were used in all-subsequent studies (Fig. 2b). Primer sets targeting mitochondrial DNA in urine, and SSU rRNA in *Ae. aegypti* mosquitoes; as positive controls, yielded similar pattern of LAMP amplicons in gel electrophoresis (Additional file 2: Figure S1 and Additional file 3: Figure S2).

Gel electrophoresis requires, of course, expert personnel not only to run the gel, but also to prevent forward contamination, arising if amplicons from an earlier assay contaminate later samples, leading to false positives. Here, the displaceable probe generates fluorescence that can be read through the wall of a sealed tube, which may remain sealed throughout the assay.

### Detection of viruses in urine by real-time RT-LAMP

**Fig. 3:**
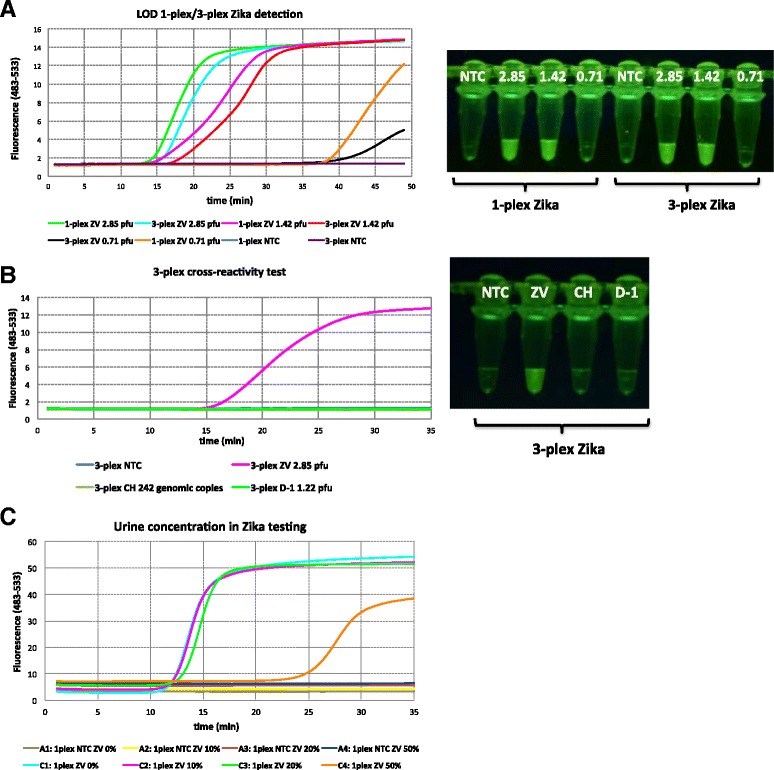
Real-time RT-LAMP on Zika detection (ZV). **a** Singleplex and multiplexed LOD for Zika using 80 nM FAM-labeled strand-displacing probe. Zika viral titers ranged from 2.85 pfu to 0.71 pfu. Fluorescent emission upon irradiation by a *blue LED* (470 nm) was visualized through *orange filter glass* after incubation at 65 °C for 30 min. **b** Cross-reactivity assay for Zika where fluorescent probes for chikungunya (HEX) and dengue-1 (TAMRA) were excluded and only FAM-labeled amplicons could be detected. Viral titers were as follows: 2.85 pfu for ZV, 242 genomic copies for CH, and 1.22 pfu for D-1. Visualization was done as before after incubation at 65 °C for 35 min. **c** Tolerance to urine in RT-LAMP. Viral titer of Zika RNA was 2.85 pfu. Varying concentrations (0%-50%) of urine were tested using 80 nM FAM-labeled strand-displacing probe

Figure 3a shows the results for the LOD for Zika using fluorescent probes targeting Zika RNA. Serial dilution study showed a clean sigmoidal amplification with 2.85 pfu per assay, with a signal rise largely complete at 20-25 min, both in cases where only Zika primers were present (1-plex) or when all three primer sets were present (3-plex). Slightly less sigmoidal curves were observed with 1.425 pfu, with signal generation being substantially complete after ~30 min. When diluted further to ~0.71 pfu, a fluorescent signal was observed only after ~40 min for a singleplexed assay, and after ~50 min for the 3-plexed mixture. In parallel, limits of detection were measured to be 37.8 copies and 1.22 pfu for chikungunya and dengue-1, respectively (Additional file 4: Figure S3).

Cross-reactivity was then tested using only Zika-targeting FAM-labeled probes in the presence of the two primer sets for chikungunya and dengue-1. Again, a strong signal was seen arising within 25-30 min, but only if Zika is present; chikungunya or dengue-1 viral RNAs gave no cross-reacting signals (Fig. 3b). Last, complete Zika virus was added to an authentic human urine sample at increasing concentrations (Fig. 3c). Presumably because of its electrolytes, the LAMP signal was delayed from 15 to 35 min at the highest ratio of urine:buffer (1:1), but not substantially at lower ratios.

**Fig. 4:**
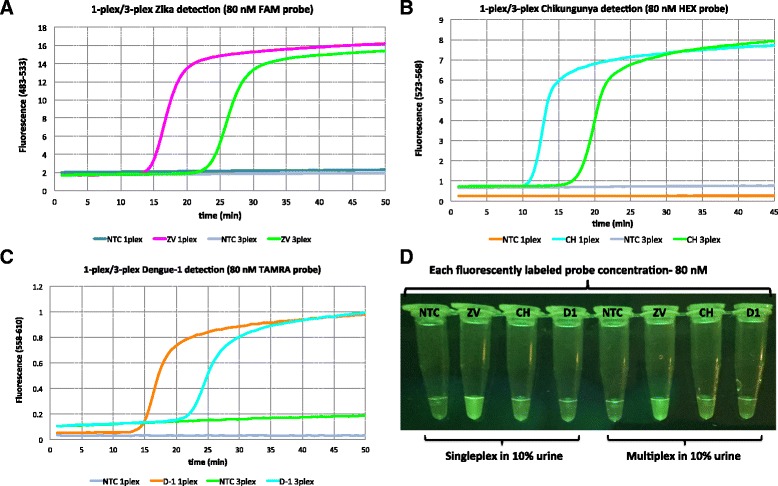
**a** Singleplex and multiplex detection of Zika (ZV) viral RNA (2.85 pfu) in 10% urine using 80 nM strand-displacing probe using Roche Light cycler (channel 483-533), **b** Singleplex and multiplex detection of chikungunya (CH) viral RNA (242 copies) in 10% urine using 80 nM strand-displacing probe using Roche Light cycler (channel 523-568), **c** Singleplex and multiplex detection of dengue-1 (D1) viral RNA (1.22 pfu) in 10% urine using 80 nM strand-displacing probe using Roche Light cycler (channel 558-610), **d** Fluorescent emission upon irradiation by a *blue LED* (470 nm) was visualized through *orange filter glass*. Each virus was assigned to a different fluorophore tag; FAM for Zika, HEX for chikungunya, and TAMRA for dengue

Strand displacing probes were then introduced to RT-LAMP that allowed the three viruses to signal with different fluorescent species: fluorescein (FAM) for Zika, HEX for chikungunya, and TAMRA for dengue. Complete viral RNAs extracted from cell cultures were used as targets, and these were presented in 10% urine. Real time analysis of the emergence of fluorescence showed a substantial difference for each target (about 10 min delays) in the 1-plex curves (where the only primers were specific for the target virus) and the 3-plex curves (where primers for all target viruses are present). However, in all cases, signal generation was effectively complete by 30 min (Fig. 4a-c). The fluorescence was observed through an orange filter upon excitation by a blue LED emitting it 470 nm. This led to different signal strengths based on the different photophysics of the three fluorophores. Thus, the FAM signal was the strongest, as the 470 nm excitation light is closest to the maximum of the FAM excitation spectrum (Fig. 4d).

**Fig. 5:**
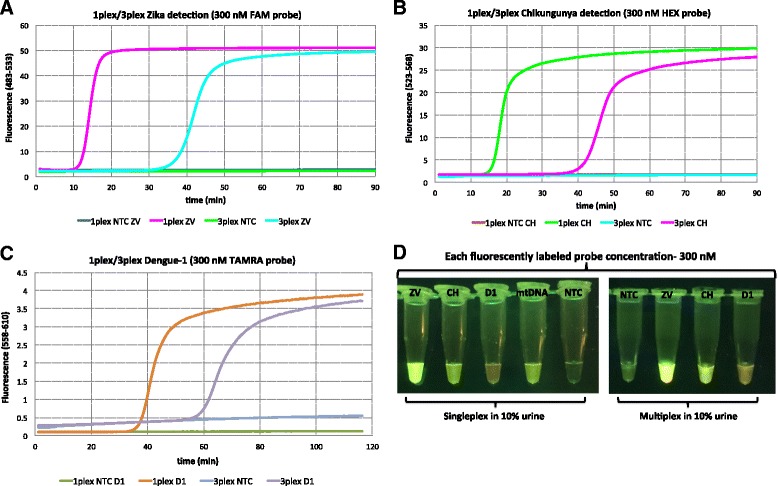
**a** Singleplex and multiplex detection of Zika (ZV) viral RNA (2.85 pfu) in 10% urine using 300 nM strand-displacing probe using Roche Light cycler (channel 483-533), **b** Singleplex and multiplex detection of chikungunya (CH) viral RNA (242 copies) in 10% urine using 300 nM strand-displacing probe using Roche Light cycler (channel 523-568), **c** Singleplex and multiplex detection of dengue-1 (D1) viral RNA (1.22 pfu) in 10% urine using 300 nM strand-displacing probe using Roche Light cycler (channel 558-610), **d** Fluorescent emission upon irradiation by a *blue LED* (470 nm) was visualized through *orange filter glass*. **mtDNA** (human mitochondrial DNA) served as a positive control (TET-labeled strand-displacing probe). Each virus was identified with a different florescent tag, FAM for Zika, HEX for chikungunya, and TAMRA for dengue

To visualize all three colors without changing the LED, the amounts of probes were increased from 80 nM to 300 nM. The results are shown in Fig. 5a-c. However, increase in the probe concentration resulted in ca. 20 min delay to obtain fluorescent signal in the 3-plexed format. In all cases, FAM-labeled probes for Zika were visualized as bright green, HEX-labeled probes for chikungunya were visualized as green-yellow, and TAMRA labeled probes for dengue-1 were visualized as orange when excited with blue LED (470 nm) and filtered through orange glass (Fig. 5d).

### Q-paper based RT-LAMP on infected mosquito samples

**Fig. 6:**
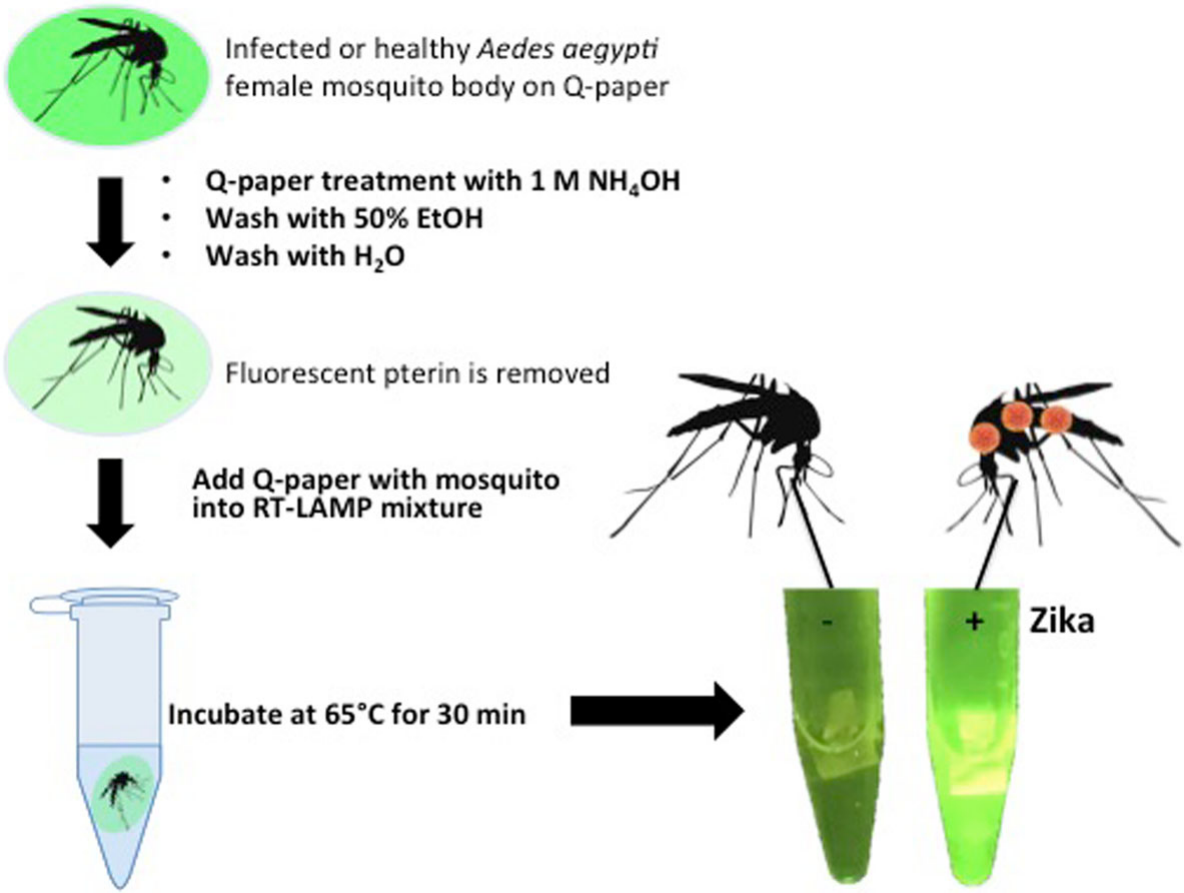
Workflow for Zika detection on infected mosquito samples using Q-paper technology. A mosquito body (ZV 9, Table 3) was first crushed on Q-paper and treated with 1 M aqueous NH_3_ (pH ≈ 12) solution, and paper was then sequentially washed with 50% EtOH and water. Q-paper containing mosquito sample were then dipped into RT-LAMP mixture and incubated at 65 °C for 30 min and fluorescent signal generated was visualized using LED *blue light* (470 nm) through *orange filter glass*. The image was recorded by cell phone camera

As a part of mosquito surveillance, a square of Q-paper carrying mosquito carcasses infected with Zika or chikungunya (Table 3), following washing with ammonia and ethanol, could be directly introduced into the LAMP mixture without negative effect (Fig. 6). Again, visual fluorescence signal was generated within 30 min (Additional file 5: Figure S4).

## Discussion

The recent Zika outbreak shows the importance of timely diagnosis of viral diseases at places where patients present with symptoms (points of sampling). It also shows the challenges faced by public health service staff as they survey the environment for mosquitoes that might be carrying arboviruses. In neither case do the practitioners want to send a sample away and wait for results.

The kit developed here provides the needed capabilities in both settings. RT-LAMP, run at 65 °C, proved to be especially convenient, as Zika and other viruses loses all infectivity at temperatures higher than 60 °C [29]. Further, RT-LAMP is shown to tolerate many low molecular weight substances in biological samples [30]. This allows viral RNAs to be LAMP-amplified without a previous RNA extraction or purification step.

For Zika, the level of virus in the urine of a patient having a current infection is well above the limits of detection (LODs) possible in this assay. Further, the level of virus in an infected mosquito capable of transmitting the virus are also well above the LOD’s reported here. The relevant levels in urine of dengue and chikungunya are also detectable in these assays [31, 32]. Therefore, the sensitivity of this kit is appropriate for all three pathogens.

To make the assay easy to use, pre-prepared tubes containing lyophilized reagents were distributed with an observation box that uses a 470 nm emitting LED and an orange filter (Additional file 6: Figure S5). This is optimal for the fluorescein (FAM) fluorophore, used here to tag Zika amplicons. It is less optimal for the HEX and TAMRA fluorophores that were used for chikungunya and dengue, respectively. Thus, the last two viruses are less easily detected by human eye than Zika, even though the amplification process appears to be no different.

It is known that some intercalating dyes (e.g. SYBR Green I) may inhibit the LAMP or PCR reactions [33]. Delay in the signal generation might be attributed to higher probe concentration, especially in the 3-plexed reactions. In one case, however, when the amount of Bst 2.0 DNA polymerase was doubled, improvement on the time to signal was observed (data not shown). On the other hand, increase in the probe concentration led to visualization of fluorescent signal generated by the presence of each target virus.

Alternatively, delays in the signal generation might be mitigated by addition of two new LEDs that have emissions more appropriate to excite the two other fluorophores. This will be necessary if higher multiplexing is desired to pick up additional arboviruses such as o’nyong nyong and Mayaro. Such higher multiplexing may also require the use of strategically placed alternative nucleic acid analogs, such as the self-avoiding molecular recognition systems described in the 22-plex for arboviruses reported by Glushakova et al. [34].

In addition to having an architecture that never requires the assay tube to be opened after the target RNA is amplified, forward contamination was mitigated by a second expedient. This replaced dTTP by a mixture of dTTP and dUTP, leaving to 2′-deoxyuridine being incorporated into the amplicons. This makes the amplicons the targets for destruction by a uracil-DNA glycosylase (UDG). Thus, thermolabile UDG digests any surviving amplicons at room temperature as the LAMP samples are being set up, preventing yesterday’s amplicons from being today’s contaminants. Further, to deploy this kit in low resource locations, any glycerol present in commercially acquired enzymes was removed by ultrafiltration, a solution containing dNTPs and LAMP primers was added, and the mixture was freeze-dried in tubes that were rehydrated on location to run the assay.

According to the literature, these pathogens can also be detected in saliva and plasma. To test this kit with these biofluids, samples of plasma and saliva were spiked with Zika viral RNA and added to the mixture in a 1:9 ratio of sample:buffer (Additional file 7: Figure S6). This work was repeated in India using plasma samples from patients infected with chikungunya and dengue exploiting the lyophilized reagent kit shipped without refrigeration. Fluorescent signal was successfully generated within 30 min (Additional file 8: Figure S7).

Another need for immediate detection involves mosquito surveillance. For example, in Haiti, when a household is found to contain an individual infected with the virus, mosquitoes in and around that household are routinely collected. These usually have lower priority for public health resources, so an inexpensive multiplexed kit to survey them would have special value. Thus, we asked whether this kit would work on mosquito carcasses that had been crushed on paper containing high levels of covalently immobilized quaternary ammonium salts (Q-paper). Q-paper has the advantage of capturing all the nucleic acids even if the mosquito carcass is treated with a sterilant (such as aqueous ammonia) or washed (for example, with ethanol, to remove fluorescent compounds from the mosquito carcass, such as pterins [35, 36], which might interfere with in-tube analysis). We were concerned that the Q-paper would itself inhibit RT-LAMP. However, we found that appropriately sized Q-paper carrying mosquito carcasses could be directly introduced into RT-LAMP mixture, and the amplification was still successful (Additional file 9: Figure S8).

## Conclusions

A kit, complete with a visualization apparatus and refrigeration-free sample transport, is now available for rapid point-of-sampling detection of Zika, chikungunya, and dengue viruses. By directly adding virus contaminated urine, plasma samples or squares of Q-paper containing infected mosquitoes to RT-LAMP mixtures, a minimum detection levels of ~0.71 pfu equivalent viral RNAs for Zika, ~1.22 pfu equivalent viral RNAs for dengue, and ~38 copies of chikungunya viral RNA, were achieved. The assay is read in 20-40 min by visualizing (human eye) three-color coded fluorescence signals. When pre-mixed reagents and enzymes are lyophilized in the tubes to be used in the assays, the tubes can be distributed to lower resource settings without refrigeration.

## Additional files

Additional file 1: Document_Point of Sampling Detection of Zika Virus within a Multiplexed Kit Capable of Detecting Dengue and Chikungunya (DOCX 111 kb)

Additional file 2: Figure S1. Gel electrophoresis of LAMP primers tested for human mitochondrial DNA in urine. No template controls (NTCs) were performed in the absence of urine sample, 1-plex or 3-plex NTCs showed no ladder like amplicons. In the presence of 10% urine, all primer sets both in 1-plex and 3-plex formats, gave ladder like amplicons (JPEG 33 kb)

Additional file 3: Figure S2. Gel electrophoresis of RT-LAMP primers tested on small subunit rRNA of female *Ae. aegypti* mosquitoes. Crushed specimens were either put directly into RT-LAMP mixture, or first crushed on Q-paper and then went through ammonia treatment prior to RT-LAMP. In either case, set 2 failed to go to completion where as for set 1, most of the primers were consumed within 30 min of incubation at 65 °C. No template control experiments did not produce any amplicon as expected (JPEG 44 kb)

Additional file 4: Figure S3. Limit of detection for 1-plex chikungunya and dengue-1 RT-LAMP experiments. Substrates for this experiment were extracted viral RNA from Vero cell cultures. **(A)** Varying titers of chikungunya viral RNAs (~189 to 18 copies) were included in RT-LAMP reagents and run real-time using Light cycler (channel 523-568). For chikungunya detection, 80 nM of HEX-labeled probes were used, and about 38 copies of chikungunya viral RNA could be detected in less than 30 min. **(B)** Varying titers of dengue-1 viral RNAs (~2.44 to 0.12 pfu equivalent RNA copies) were included in RT-LAMP reagents and run real-time using Light cycler (channel 558-610). For dengue-1 detection, 80 nM of TAMRA-labeled probes were used, and about 1.22 pfu equivalent copies of dengue-1a viral RNA could be detected within 35 min (JPEG 58 kb)

Additional file 5: Figure S4. Gel-electrophoresis and visualization of RT-LAMP products with LED blue light (excitation at 470 nm) through orange filter. **(A)** Detection of Zika (ID # 3 and 4) and chikungunya (ID # 320 and 328) in 3-plex format with infected mosquito legs or bodies. Zika infected mosquitos generated bright green fluorescence (FAM-labeled probe) whereas chikungunya infected mosquitoes generated yellow-green fluorescence (HEX-labeled probe). Gel electrophoresis analysis showed that in the presence of target viral RNA, ladder like amplicons were generated. **(B)** Visualization of Zika-infected (ID # 7 and 9) and chikungunya-infected (ID # 191) mosquito samples in 3-plex format on Q-paper after RT-LAMP run at 65 °C for 30 min. Zika samples generated bright green signal due to FAM-labeled probes whereas chikungunya containing samples generated more like yellow-green signal due to the use of HEX-labeled probes (JPEG 56 kb)

Additional file 6: Figure S5. This observation box is now available for point of sampling rapid detection of Zika, chikungunya, and dengue. This box uses a 470 nm emitting LED blue light and an orange filter with a single AA battery already embedded (JPEG 40 kb)

Additional file 7: Figure S6. Gel electrophoresis analysis of Zika detection in saliva and blood. Like urine RT-LAMP experiments, extracted Zika viral RNAs (2.85 pfu) were spiked with saliva and plasma samples, and 10% final concentration of saliva or plasma was included into RT-LAMP mixtures. Zika positive samples were identified as ladder-like amplicons on agarose gel (JPEG 21 kb)

Additional file 8: Figure S7. **(Top)** Real-time RT-LAMP of chikungunya samples using Rotor Gene Q (Qiagen, Germantown, MD, USA). Using dry format RT-LAMP, 9 plasma samples and 1 purified RNA sample were tested in real-time and fluorescent signals were generated within 30 min for all cases. **(Bottom)** RT-LAMP on dengue samples (plasma) was tested and signal generation was observed by detection box from Additional file 6: Figure S5 (JPEG 46 kb)

Additional file 9: Figure S8. Gel-electrophoresis of RT-LAMP primers tested on Zika or chikungunya infected female *Ae. aegypti* mosquitoes (Table 3) crushed on Q-paper and went through ammonia treatment. Zika-infected *Ae. aegypti* (ID # 7) and chikungunya-infected *Ae. aegypti* (ID # 378) samples on Q-paper were run in 1-plex format whereas chikungunya infected mosquito (ID # 401) was run in 3-plex format where all primers for Zika, chikungunya and dengue-1 were present in the RT-LAMP mixture. All samples with presented virus were able to generate ladder like amplicons within 30 min of incubation at 65 °C (JPEG 38 kb)

## Abbreviations

LAMP: Loop-mediated isothermal amplification
LOD: Limit of detection
MSAs: Multiple sequence alignments
Q-paper: Quaternary ammonium functionalized Whatman paper
RT-LAMP: Reverse transcription loop-mediated isothermal amplification
SSU rRNA: small subunit ribosomal RNA
UDG: Uracil DNA glycosylase
