# Supplementary material for: Point of sampling detection of Zika virus within a multiplexed kit capable of detecting dengue and chikungunya
Source: BMC Infect Dis. 2017 Apr 20;17:293. doi: 10.1186/s12879-017-2382-0 (PMC5399334; doi:10.1186/s12879-017-2382-0)
Supplement: Supplementary file 1 — Document_Point of Sampling Detection of Zika Virus within a Multiplexed Kit Capable of Detecting Dengue and Chikungunya (DOCX 111 kb) [file 12879_2017_2382_MOESM1_ESM.docx]

Supplementary Materials for “**Point of Sampling Detection of Zika Virus within a Multiplexed Kit Capable of Detecting Dengue and Chikungunya**”

**1. Additional primers and probes for RT-LAMP experiments**

Primers and probes represented in **Suppl. Table 1** were tested, and some sets found to be performing not as good as those shown in the main manuscript. Set 3 and set 4 for human mitochondrial DNA in urine have shown to be working just fine, but only set 1 was used in control experiment (**Suppl. Figure 1**).

For mosquito control experiments, LAMP reactions with primer set 1 targeting small subunit rRNA were successfully conducted both in solution and Q-paper. However, set 2 failed to produce successful amplicons. Therefore, this set has been abandoned (**Suppl. Figure 2**).

**Suppl. Table 1**. Other LAMP primers targeting human mitochondrial DNA and Aedes aegypti small subunit rRNA

| **Target virus (targeted gene)** | **Name** | **Sequence (5’-3’)** | **Length** | **Start Pos** | **End Pos** |
| --- | --- | --- | --- | --- | --- |
| **Human (mitochondrial DNA, set3)** | MtDNA3-F3 | CTCTTACCTCCCTCTCTC | 18 | 6240 | 6257 |
|  | MtDNA3-B3 | GGTATTGGGTTATGGCAG | 18 | 6468 | 6451 |
|  | MtDNA3-LB | TCCTTACACCTAGCAGGTG | 19 | 6375 | 6393 |
|  | MtDNA3-LF | CTGTTCAACCTGTTCCTGC | 19 | 6312 | 6294 |
|  | MtDNA3-FIP | GGAGTAGTTCCCTGCTAAGGTTTTTTGCTATAGTGGAGGCC | 41 | 6275 | 6341 |
|  | MtDNA3-BIP | CCTCCGTAGACCTAACCATCTTTTTGCCCCTAAGATAGAGGAG | 43 | 6352 | 6412 |
|  | MtDNA3-LB_NatTail | **TET**-CGGGTTTGCGCTCAGCCATCCGTTCAGTCCGTCAGGTCAG TCCTTACACCTAGCAGGTG | 59 | 6375 | 6393 |
| **Human (mitochondrial DNA, set4)** | MtDNA4-F3 | CGGCTTACTTCTCTTCC | 17 | 15499 | 15515 |
|  | MtDNA4-B3 | GGATATATGGAGGATGGG | 18 | 15739 | 15722 |
|  | MtDNA4-LB | CCGTCCCTAACAAACTAGGAG | 21 | 15657 | 15677 |
|  | MtDNA4-LF | GGTATAATTGTCTGGGTCGC | 20 | 15580 | 15561 |
|  | MtDN4-FIP | GGTGTTTAAGGGGTTGGCTTTTTCACTATTCTCACCAGACC | 41 | 15537 | 15601 |
|  | MtDNA4-BIP | CGCCTACACAATTCTCCGTTTTTGTAATAGGGCAAGGACG | 40 | 15637 | 15695 |
|  | MtDNA4-LB_NatTail | **TET**-  CGGGTTTGCGCTCAGCCATCCGTTCAGTCCGTCAGGTCAG CCGTCCCTAACAAACTAGGAG | 61 | 15657 | 15677 |
|  | Common quencher | CTGACCTGACGGACTGAACGGATGGCTGAGCGCAAACCCG-**Iowa Black FQ** | 40 |  |  |
| ***Aedes aegypti* (SSU rRNA) set2** | Aae2-F3 | TCGTATGGTCTCGCAC | 16 | 356 | 375 |
|  | Aae2-B3 | TTCTCGTCACTACCTCC | 17 | 578 | 559 |
|  | Aae2-LB | CACATCCAAGGAAGGCAG | 18 | 505 | 523 |
|  | Aae2-LF | CCCGTTACCCGTTGCA | 16 | 458 | 443 |
|  | Aae2-FIP | CCGGAATCGAACCCTGATTTTTTGATGGTAGTATAGAGGACTAC | 44 | 416 | 479 |
|  | Aae2-BIP | GAGCCTGAGAAATGGCTACTTTTTGGGATTGGGTAATTTACG | 42 | 485 | 551 |

**2. Mosquito infection with Zika and chikungunya**

Field-collected mosquito larvae from discarded containers in Florida were reared in plastic photo trays (25 cm width, 30 cm length, 5 cm height; Richard MFG Co. Fernandina Beach, FL, U.S.A) with water (900 mL) and larval food (0.4 g) consisting of equal parts of liver powder and brewer’s yeast. Supplemental food was provided 3-4 days later. Pupae were transferred to clear plastic tubes with water and sealed with a cotton lid. Mosquitoes were identified to species upon eclosion and *Ae. aegypti* were maintained in a laboratory colony. Adult mosquitoes were maintained in a 0.3^3^ m cage (laboratory colony) with access to 10% sucrose and water from cotton wicks in a climate-controlled room at 28°C and a 12:12-hour light : dark cycle. Weekly blood meals were provided to mosquitoes from chickens. Males and females are held within the same cage to ensure females are mated. Eggs were collected on paper towels that lined cups with water held within the cages. The F_1-3_ progeny *Ae. aegypti* mosquitoes were used in the infection experiment.

Adult females aged 7-10 days were deprived of sucrose for one day and then allowed to feed on virus infected blood using an artificial membrane feeding system (Hemotek, Discovery Workshops, Accrington, UK). Virus stocks and infectious blood meals were prepared by inoculating monolayers of Vero cells (T175 flasks) with approximately 0.1 multiplicity of infection of either Zika and chikungunya viruses. Tissue culture flasks with cells were incubated for one hour at 37°C and 5% CO2 atmosphere to allow for virus attachment and entry, after which 25 ml of media (M199 medium supplemented with 10% fetal bovine plasma, penicillin/streptomycin and mycostatin) were added. Flasks infected with each virus were then incubated at 37°C and 5% CO_2_ atmosphere: Chikungunya virus, three days and Zika viruses, seven days. Female mosquitoes were held in cylindrical cages (h by d: 10 cm by 10 cm) with mesh screening from which they ingested the infected blood during one hour feeding trials. Viral titers in the infected blood meals were as follows: 7.0 log10 plaque forming units (pfu)/mL for Zika virus, 8.2 log10 pfu/mL for the Indian Ocean lineage of Chikungunya virus, and 8.4 log10 pfu/mL for the Asian lineage of Chikungunya virus). Following feeding trials, fully engorged females were held in cylindrical cages with 10% sucrose and an oviposition substrate in an incubator (12:12-hour light : dark cycle and 30°C) in the Biosafety level-3 facility at the Florida Medical Entomology laboratory in Vero Beach, FL. Aliquots of blood were stored at -80°C for later determination of viral titer of blood ingested by mosquitoes. Cohorts of mosquitoes were tested for transmission of chikungunya virus at 2, 5-6, and 12-13 days and then killed and stored at -80°C as part of an unrelated study. Mosquitoes that ingested Zika viruses were held for 14 days and then killed and stored at -80°C. Subsamples of these mosquitoes were used in the project described in this paper.

Mosquitoes were individually dissected to remove the legs from the bodies. The legs, used for controls, were tested for viral RNA presence. Primers and probes used for viral RNA detection of Zika and chikungunya viruses were represented in **Suppl. Table 2** using the Superscript III One-Step qRT-PCR with Platinum® Taq kit by Invitrogen (Invitrogen, Carlsbad, CA) as described previously [[1](#_ENREF_1)] with the CFX96 Real-Time PCR Detection System (Bio-Rad Laboratories, Hercules, CA)

**Suppl. Table 2**. Primers and probes for viral RNA detection

| **Target virus** | **Target gene** | **Name** | **Sequence (5’-3’)** |
| --- | --- | --- | --- |
| **Zika** | NS5 gene | Forward primer | CTTCTTATCCACAGCCGTCTC |
|  |  | Reverse primer | CCAGGCTTCAACGTCGTTAT |
|  |  | probe | FAM-AGAAGGAGACGAGATGCGGTACAGG-BHQ-1 |
| **Chikungunya** | nonstructural polyprotein gene common to both lineages | Forward primer | GTACGGAAGGTAAACTGGTATGG |
|  |  | Reverse primer | TCCACCTCCCACTCCTTAAT |
|  |  | probe | FAM-TGCAGAACCCACCGAAAGGAAACT-BHQ-1 |

**3. Limit of detection studies for chikungunya and dengue-1 viruses**

Varying titers of each virus was included in RT-LAMP mixtures and fluorescence was recorded in real-time using Roche Light cycler 480. **(Suppl. Figure 3)**

**4. Detection of Zika and chikungunya on infected mosquitoes**

**Suppl. Figure 4** depicts gel-electrophoresis and visualization of RT-LAMP products with LED blue light (excitation at 470 nm) through orange filter.

**5. Signal detection component of point-of-care diagnostics**

In house built observation box was used as a detection component in this assay. **(Suppl. Figure 5**)

**6. Detection of Zika virus in unprocessed saliva and plasma samples**

Gel electrophoresis analysis of Zika viral RNA detection in saliva and blood was shown in **Suppl. Figure 6**.

**7. RT-LAMP on plasma samples from patients infected with chikungunya and dengue**

**Suppl. Figure 7** shows real-time analysis of RT-LAMP on chikungunya infected plasma samples and signal generation by observation box on dengue infected plasma samples.

**8. Gel electrophoresis analysis of Zika and chikungunya infected mosquitoes on Q-paper**

Gel-electrophoresis analysis of RT-LAMP on Zika or chikungunya infected female *Ae. aegypti* mosquitoes (Table 3) using Q-paper method. **(Suppl. Figure 8)**

1. Reiskind MH, Pesko K Fau - Westbrook CJ, Westbrook Cj Fau - Mores CN, Mores CN: **Susceptibility of Florida mosquitoes to infection with chikungunya virus**. *Am J Trop Med Hyg* 2008, **78**(3):422-425.
