# Supplementary figures and images for: Point of sampling detection of Zika virus within a multiplexed kit capable of detecting dengue and chikungunya
Source: BMC Infect Dis. 2017 Apr 20;17:293. doi: 10.1186/s12879-017-2382-0 (PMC5399334; doi:10.1186/s12879-017-2382-0)

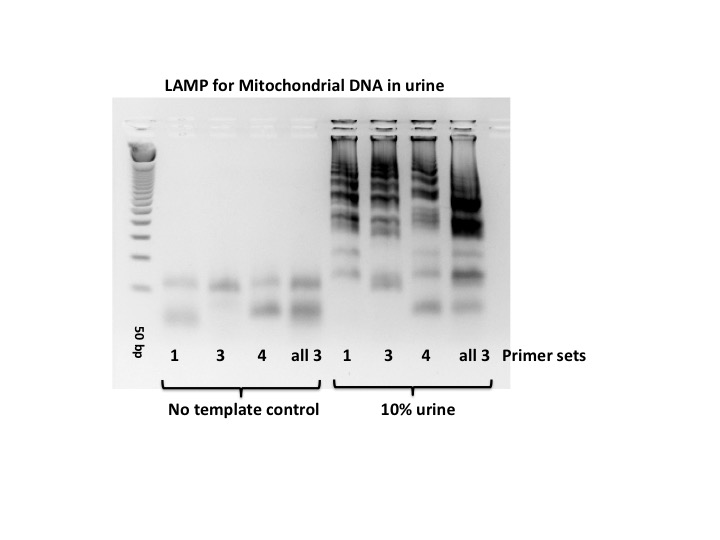

Supplement: Supplementary file 2 — Gel electrophoresis of LAMP primers tested for human mitochondrial DNA in urine. No template controls (NTCs) were performed in the absence of urine sample, 1-plex or 3-plex NTCs showed no ladder like amplicons. In the presence of 10% urine, all primer sets both in 1-plex and 3-plex formats, gave ladder like amplicons (JPEG 33 kb) [file 12879_2017_2382_MOESM2_ESM.jpg]

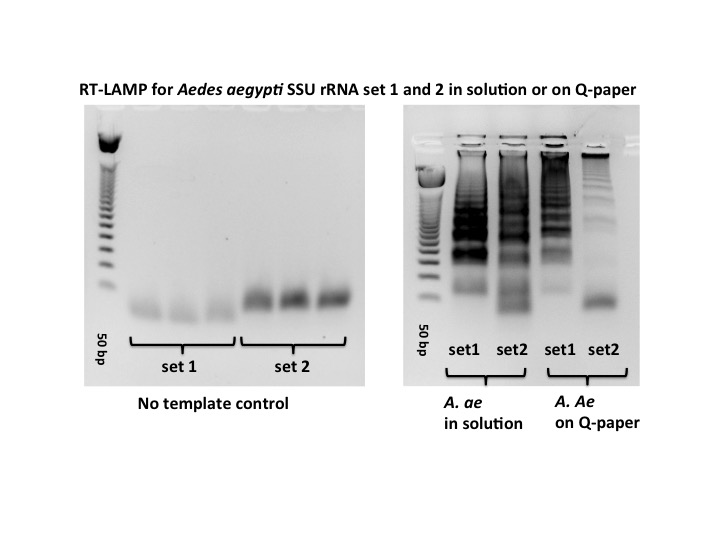

Supplement: Supplementary file 3 — Gel electrophoresis of RT-LAMP primers tested on small subunit rRNA of female Ae. aegypti mosquitoes. Crushed specimens were either put directly into RT-LAMP mixture, or first crushed on Q-paper and then went through ammonia treatment prior to RT-LAMP. In either case, set 2 failed to go to completion where as for set 1, most of the primers were consumed within 30 min of incubation at 65 °C. No template control experiments did not produce any amplicon as expected (JPEG 44 kb) [file 12879_2017_2382_MOESM3_ESM.jpg]

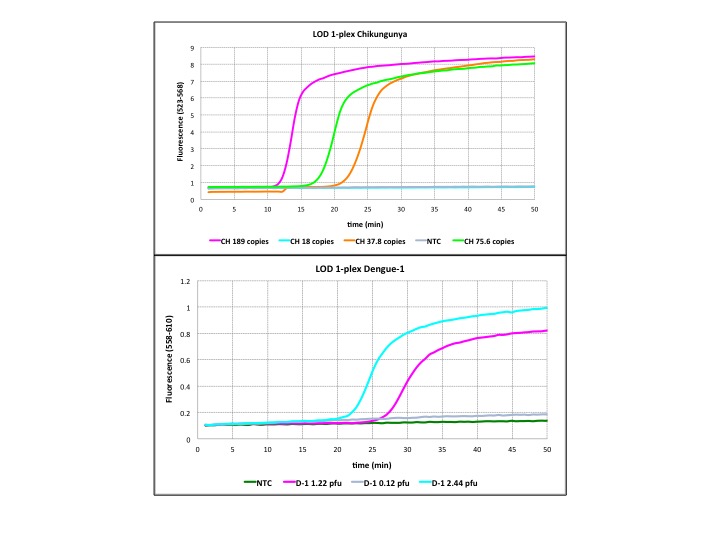

Supplement: Supplementary file 4 — Limit of detection for 1-plex chikungunya and dengue-1 RT-LAMP experiments. Substrates for this experiment were extracted viral RNA from Vero cell cultures. (A) Varying titers of chikungunya viral RNAs (~189 to 18 copies) were included in RT-LAMP reagents and run real-time using Light cycler (channel 523-568). For chikungunya detection, 80 nM of HEX-labeled probes were used, and about 38 copies of chikungunya viral RNA could be detected in less than 30 min. (B) Varying titers of dengue-1 viral RNAs (~2.44 to 0.12 pfu equivalent RNA copies) were included in RT-LAMP reagents and run real-time using Light cycler (channel 558-610). For dengue-1 detection, 80 nM of TAMRA-labeled probes were used, and about 1.22 pfu equivalent copies of dengue-1a viral RNA could be detected within 35 min (JPEG 58 kb) [file 12879_2017_2382_MOESM4_ESM.jpg]

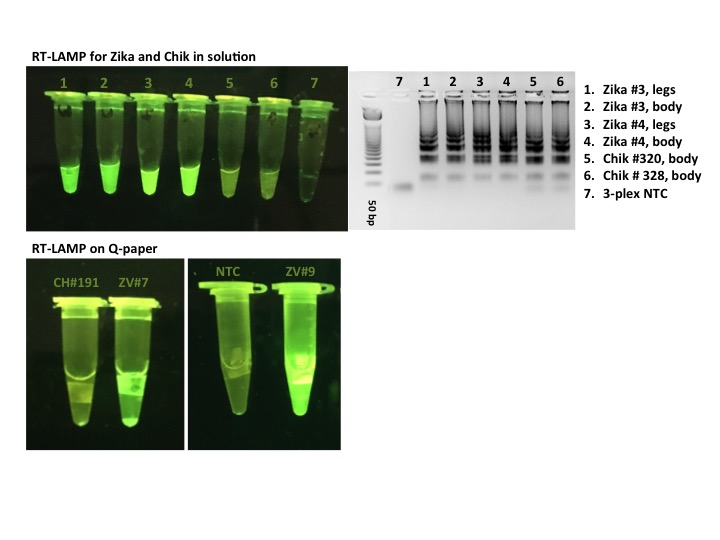

Supplement: Supplementary file 5 — Gel-electrophoresis and visualization of RT-LAMP products with LED blue light (excitation at 470 nm) through orange filter. (A) Detection of Zika (ID # 3 and 4) and chikungunya (ID # 320 and 328) in 3-plex format with infected mosquito legs or bodies. Zika infected mosquitos generated bright green fluorescence (FAM-labeled probe) whereas chikungunya infected mosquitoes generated yellow-green fluorescence (HEX-labeled probe). Gel electrophoresis analysis showed that in the presence of target viral RNA, ladder like amplicons were generated. (B) Visualization of Zika-infected (ID # 7 and 9) and chikungunya-infected (ID # 191) mosquito samples in 3-plex format on Q-paper after RT-LAMP run at 65 °C for 30 min. Zika samples generated bright green signal due to FAM-labeled probes whereas chikungunya containing samples generated more like yellow-green signal due to the use of HEX-labeled probes (JPEG 56 kb) [file 12879_2017_2382_MOESM5_ESM.jpg]

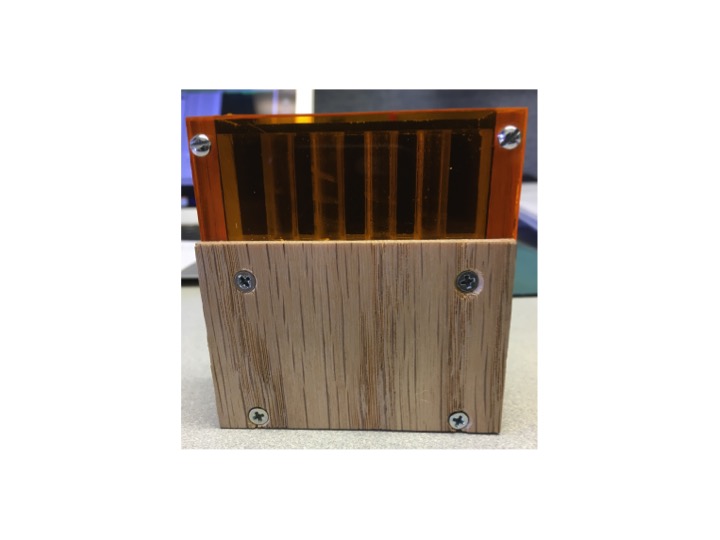

Supplement: Supplementary file 6 — This observation box is now available for point of sampling rapid detection of Zika, chikungunya, and dengue. This box uses a 470 nm emitting LED blue light and an orange filter with a single AA battery already embedded (JPEG 40 kb) [file 12879_2017_2382_MOESM6_ESM.jpg]

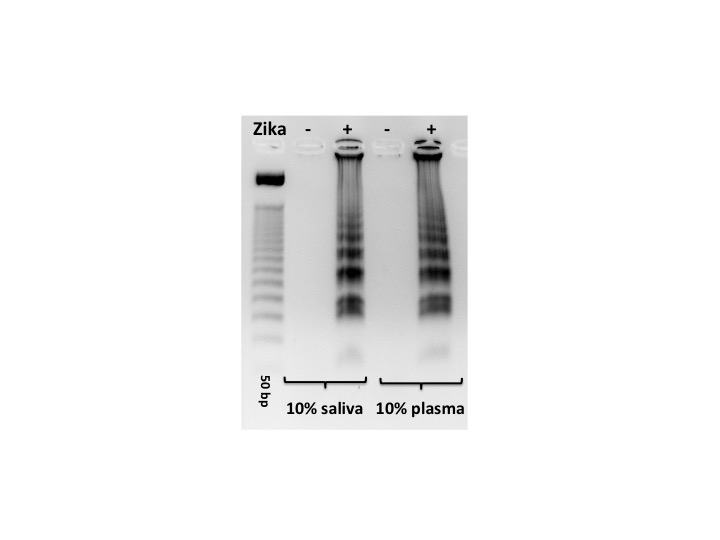

Supplement: Supplementary file 7 — Gel electrophoresis analysis of Zika detection in saliva and blood. Like urine RT-LAMP experiments, extracted Zika viral RNAs (2.85 pfu) were spiked with saliva and plasma samples, and 10% final concentration of saliva or plasma was included into RT-LAMP mixtures. Zika positive samples were identified as ladder-like amplicons on agarose gel (JPEG 21 kb) [file 12879_2017_2382_MOESM7_ESM.jpg]

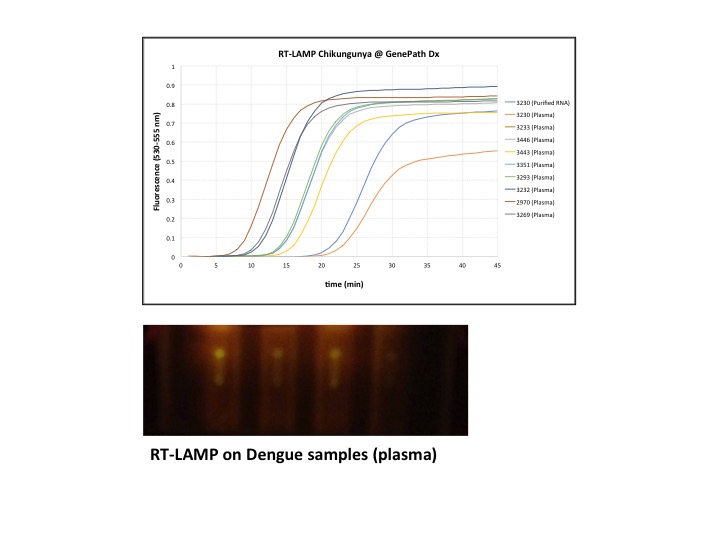

Supplement: Supplementary file 8 — (Top) Real-time RT-LAMP of chikungunya samples using Rotor Gene Q (Qiagen, Germantown, MD, USA). Using dry format RT-LAMP, 9 plasma samples and 1 purified RNA sample were tested in real-time and fluorescent signals were generated within 30 min for all cases. (Bottom) RT-LAMP on dengue samples (plasma) was tested and signal generation was observed by detection box from Additional file 6: Figure S5 (JPEG 46 kb) [file 12879_2017_2382_MOESM8_ESM.jpg]

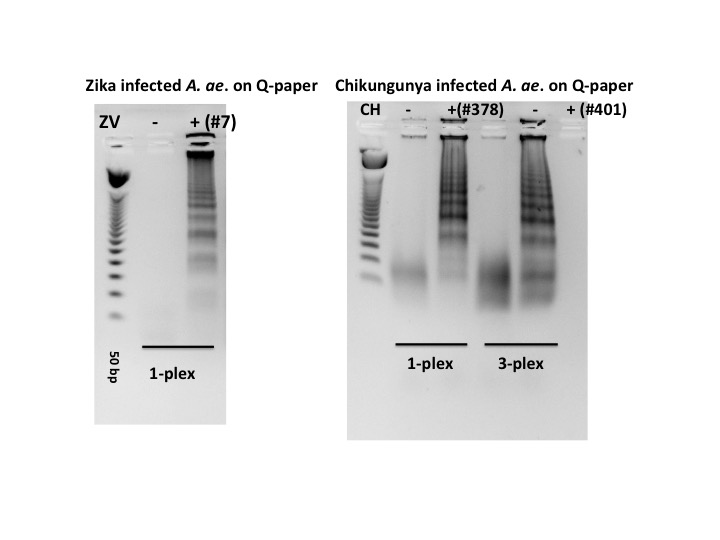

Supplement: Supplementary file 9 — Gel-electrophoresis of RT-LAMP primers tested on Zika or chikungunya infected female Ae. aegypti mosquitoes (Table 3) crushed on Q-paper and went through ammonia treatment. Zika-infected Ae. aegypti (ID # 7) and chikungunya-infected Ae. aegypti (ID # 378) samples on Q-paper were run in 1-plex format whereas chikungunya infected mosquito (ID # 401) was run in 3-plex format where all primers for Zika, chikungunya and dengue-1 were present in the RT-LAMP mixture. All samples with presented virus were able to generate ladder like amplicons within 30 min of incubation at 65 °C (JPEG 38 kb) [file 12879_2017_2382_MOESM9_ESM.jpg]
